# Characterization and thermogravimetric analysis of lanthanide hexafluoroacetylacetone chelates

**DOI:** 10.1007/s10967-016-5005-0

**Published:** 2016-08-30

**Authors:** Shayan Shahbazi, S. Adam Stratz, John D. Auxier, Daniel E. Hanson, Matthew L. Marsh, Howard L. Hall

**Affiliations:** 1 Department of Nuclear Engineering, University of Tennessee, 301 Middle Dr., Pasqua Nuclear Engineering Building, Knoxville, TN 37996 USA; 2 Radiochemistry Center of Excellence, University of Tennessee, 1508 Middle Dr., Ferris Hall, Knoxville, TN 37996 USA; 3 Institute for Nuclear Security, University of Tennessee, 1640 Cumberland Ave. Howard Baker Jr. Center for Public Policy, Knoxville, TN 37996 USA; 4 Department of Chemistry, University of Tennessee, 1420 Circle Dr., Buehler Hall, Knoxville, TN 37996 USA

**Keywords:** Nuclear forensics, Thermogravimetric analysis, Thermochromatography, Rapid separations, Hexafluoroacetylacetone, Lanthanides

## Abstract

This work reports the thermodynamic characterizations of organometallic species as a vehicle for the rapid separation of volatile nuclear fission products via gas chromatography due to differences in adsorption enthalpy. Because adsorption and sublimation thermodynamics are linearly correlated, there is considerable motivation to determine sublimation enthalpies. A method of isothermal thermogravimetric analysis, TGA-MS and melting point analysis are employed on thirteen lanthanide 1,1,1,5,5,5-hexafluoroacetylacetone complexes to determine sublimation enthalpies. An empirical correlation is used to estimate adsorption enthalpies of lanthanide complexes on a quartz column from the sublimation data. Additionally, four chelates are characterized by SC-XRD, elemental analysis, FTIR and NMR.

**Electronic supplementary material:**

The online version of this article (doi:10.1007/s10967-016-5005-0) contains supplementary material, which is available to authorized users.

## Introduction

Chemical separations of rare earth (RE) elements are essential to the development of renewable energies, hybrid vehicles, personal electronics, and nuclear security. In recent years, there has been a movement to improve the “greenness” of reactions, in particular by aiming toward solvent-free reactions [1, 2]. This is readily accomplished using gas-phase separations. A leading area of rapid gas-phase experimentation has been pioneered by groups for super heavy element discovery and analysis as described by Schädel and Shaughnessy et al. [3], Zvára [4], and recently reported by Even et al. [5]. In addition to the possibility of commercialization, there is interest in improving rapid separations for the purposes of nuclear forensics as emphasized by the Nuclear Forensics and Attribution Act (NFAA) of 2010 [6]. The NFAA has called upon the scientific community to address gaps in nuclear forensics technology for the immediate mitigation of developments in nuclear terrorism. In the event of detonation of a nuclear device, the fallout debris can be analyzed for signature isotopes that may provide evidence on the origin and type of nuclear materials used as well as other clues to the device design [7, 8]. It is therefore of interest to develop sample preparation methods that can reduce the time required to perform the separation while retaining equivalent accuracy and precision to the established methods.

One method of analysis is the rapid gas phase separation of rare earth metals from other components of the debris via isothermal thermochromatography [9]. Lanthanoids cover a portion of the fission product curve with varying yields dependent on a number of variables (e.g. neutron energy, fissile parent, etc.), motivating the chromatographic separation of these elements. Early studies have shown the utility of volatile β-diketonate ligands as separating agents for rare earth metals [10–12]. In previous work, 1,1,1,5,5,5-hexafluoroacetylacetone (hfac) has been shown to produce volatile complexes with lanthanoids [13]. In our work, the use of β-diketonates as a means of volatilization [14] for the possible commercial separations of rare earth elements will be discussed, employing hfac complexes for the entire lanthanide series (except cerium and promethium).

Isothermal thermochromatography is a gas phase separation method that uses constant column temperatures to vary retention time. The adsorption time of a compound on a column, i.e. retention time, is dependent on the thermodynamics of that compound, most notably the adsorption enthalpy for that state. Studies of heavy metals have generated empirical correlations between adsorption enthalpy on a quartz column and sublimation enthalpy of the solid [3, 15, 16].

Thermogravimetric analysis (TGA) is useful in the characterization of a compound’s thermodynamics, especially the sublimation enthalpy. Many methods analyze weight loss curves as a function of a constant heating rate [17–21]. Another method introduced by Langmuir analyzes weight loss curves over isothermal heating regions [22–25]. Work presented here focuses on the determination of the sublimation enthalpies of thirteen lanthanide hfac complexes via the latter method henceforth described as the Langmuir method. Additionally, there is investigation into the degradation of the compound via constant heating TGA up to higher temperatures. This provides information on the onset temperatures of degradation, as well as the peak temperature at which total sublimation occurs. Visual melting point analysis is carried out to confirm sublimation is occurring prior to melting. Finally, characterization including SC-XRD, elemental analysis, FTIR and NMR is employed for four of the resulting compounds: Sm[hfac]_4_ (**1**), Gd[hfac]_4_ (**2**), Dy[hfac]_4_ (**3**), and Tm[hfac]_4_ (**4**).

## Theory

The sublimation enthalpy of a particular compound is a measure of its volatility. This parameter can be found many ways, but is typically measured by a method of thermogravimetric analysis [17–20]. TGA typically consists of temperature gradients applied to samples in an isobaric environment, and some studies have shown some success with finding the activation enthalpy of a compound over its various stages of decomposition [21]. Yet this experiment used multiple isothermal environments each over a finite time period instead. Langmuir has described a method of vapor pressure determination for compounds under vacuum [25]. Recently, Ashcroft has used this method which uses isothermal mass loss rates for less volatile or less thermally stable species with equally rapid measurements and analysis [22]. This has been recently employed for similar complexes, including some metal β-diketonate complexes [23, 24].

### Langmuir method

Sublimation and other phase changes follow zero-order kinetics, therefore the rate of mass loss by sublimation (*m*_sub_) is constant at a given temperature with a change in time (Δ*t*).

1 $$m_{\text{sub}} = \frac{{\Delta [{\text{mass}}\;{\text{of}}\;{\text{sample}}]}}{\Delta t}$$

**Fig. 1:**
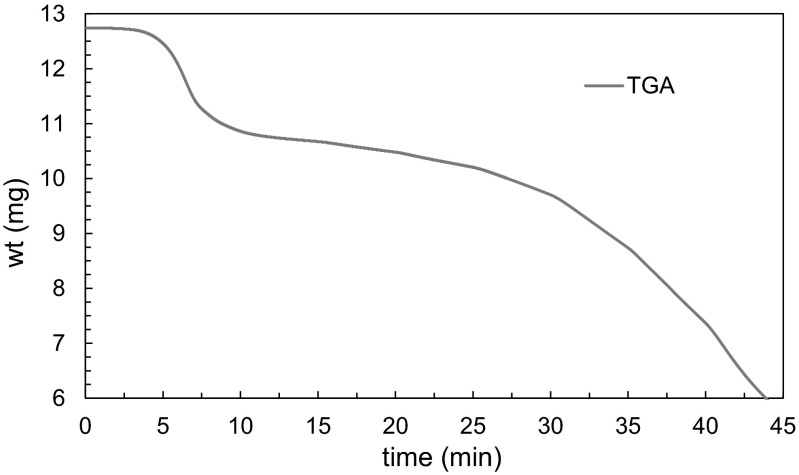
Sample mass loss curve for La[hfac]_4_

This linear mass loss rate of the sample is determined for each isotherm via TGA data. Figure 1 displays example mass loss data for each isotherm of one sample, jumping in temperature every 5 min.

Langmuir related the vapor pressure of a solid, *p*, with its sublimation rate, *m*_sub_, where *T* is the temperature, *R* is the gas constant, *α* is the vaporization coefficient (usually assumed to be 1 when under vacuum) and *M*_W_ is the molecular weight of the compound [25]. When not under vacuum, the vaporization coefficient cannot assumed to be unity, and must be found via calibration to a standard of known vapor pressure [26].

2 $$p = \sqrt {\frac{2\pi RT}{{\alpha^{2} M_{\text{W}} }}m_{\text{sub}} }$$

The Clausius–Clapeyron relation may be written as Eq. (3), where Δ*H*_sub_ is the enthalpy of sublimation.

3 $$\frac{{{\text{d}}(\ln p)}}{{{\text{d}}T}} = \frac{{\Delta H_{\text{sub}} }}{{RT^{2} }}$$

Combining Eqs. (2) and (3) and integrating yields a correlation between the mass loss rate and the enthalpy of sublimation. The resulting Eq. (4) can be written in a linear form.

4 $$\ln \left( {m_{\text{sub}} \sqrt {\frac{T}{{M_{\text{W}} }}} } \right) = \frac{{ - \Delta H_{\text{sub}} }}{RT} + \left[ {\frac{{\Delta H_{\text{sub}} }}{{RT_{\text{sub}} }} - \ln \left( {\sqrt {\frac{2\pi R}{{\alpha^{2} }}} } \right)} \right]$$

A plot of the left hand side of Eq. (4) versus 1/*T* provides a linear trend line with slope used to find the enthalpy of sublimation. The y-intercept of such line can then be used to determine the vapor pressure of the compound based on specific value of *α*. It is worth noting that not using vacuum conditions has no effect on the value of enthalpy found because the vaporization coefficient is dependent on experimental setup and not of the substance, assuming the vapor is not associating. Therefore its value does not change for each material, but only for each experimental setup, which stays constant here.

### Correlation to adsorption thermodynamics

The adsorption enthalpy of a compound is a useful parameter in gas phase chromatography. Compounds of varying adsorption enthalpy will spend different amounts of time on a chromatography column, and can be identified by their retention times. Various studies have shown thermodynamic or kinetic models that relate the adsorption enthalpy of a compound to its retention time in a gas chromatographic (GC) column [9, 27, 28]. One such example is a Monte Carlo thermochromatography (MCTC) model which utilizes the adsorption enthalpy of a compound to estimate the retention time through a GC column. This is achieved by single atom studies which simulate the movement of a random atom based on kinetic relationships [28].

At present time, there is no correlation of sublimation enthalpy to adsorption enthalpy for Ln[hfac]_4_ complexes, therefore this is a goal of future work. However there are many studies correlating the adsorption enthalpy of heavy metal compounds on quartz surfaces to the sublimation enthalpy [3]. A correlation for material with similar volatility is that for the metal chlorides or oxychlorides on a quartz column.

5 $$- \Delta H_{\text{ads}} = \left( {21.5 \pm 5.2} \right) + \left( {0.600 \pm 0.025} \right)\Delta H_{\text{sub}}$$

Using (5), the adsorption enthalpy can be estimated and used to estimate the retention time in gas phase separations as noted above.

## Experimental

### Materials

**Fig. 2:**
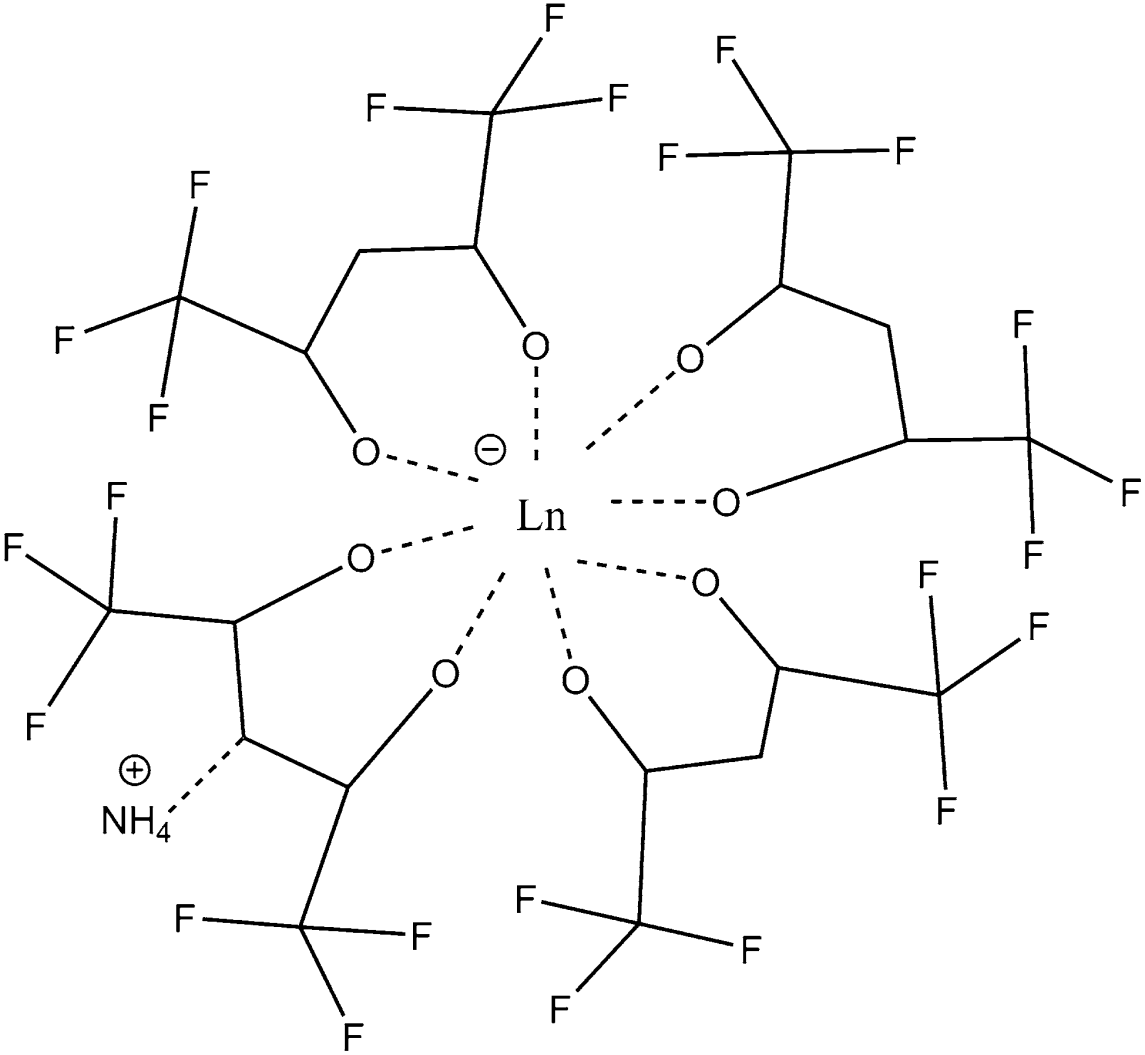
NH_4_·Ln[hfac]_4_, where Ln is La, Pr, Nd, Sm, Eu, Gd, Tb, Dy, Ho, Er, Tm, Yb or Lu

The β-diketonate complexes were produced at the Radiochemistry Center of Excellence within the Institute for Nuclear Security at the University of Tennessee (Knoxville, TN, USA). The complexes synthesized were NH_4_·Ln[hfac]_4_, where Ln is the following: La, Pr, Nd, Sm, Eu, Gd, Tb, Dy, Ho, Er, Tm, Yb or Lu, as seen in Fig. 2. Hfac complexes of Ce or Pm were not available. Synthesis and purification followed that done by Hanson et al. [29] and other literature reports [30–35]. All reagents and solvents were used from commercial sources and used without further purification. The RE oxides (Sigma Aldrich, 99.99 %) were combined with hot, concentrated HCl (Fisher, ACS Reagent Grade) to yield the chloride salt. The solution was allowed to cool. 1,1,1,5,5,5-hexafluoroacetylacetone (H[hfac], Acros, 99.9 %) was obtained and combined with equimolar amounts of concentrated NH_4_OH (Fisher, ACS Reagent Grade) at 0 °C. The two liquids reacted vigorously producing a white solid (NH_4_[hfac]) that was stirred to fully react the reagents. The solid was then placed in a desiccator for storage. The NH_4_[hfac] was dissolved in 5 mL of diethyl ether (ACS Reagent Grade, Fisher) to which the aqueous RE chloride was added in a ratio of 4:1. The mixture was shaken vigorously for 30 s, and then set for 5 min, repeating 3 times. At the conclusion of the last agitation, the organic phase was drawn off and placed in a vacuum desiccator to dry the sample and remove the ether (Fig. 2).

### Methods

A Q50 TGA (TA Instruments, USA) was used to perform all isothermal experiments related to the determination of the sublimation enthalpy via the Langmuir method. A nitrogen gas flow at 60 mL min^−1^ was used at below atmospheric pressure. Although some TGA instruments restrict sample sizes as low as 2 mg, this TGA allowed sample sizes between 10 and 15 mg, which is necessary to maintain constant surface area. The same open faced High Temperature Platinum (HT Pt) pan was used for all samples, and tared following flame torch cleanings between runs. The sample was dried in open air for 1 h and loaded in the center of the pan in a circular fashion. The diameter of the sample is estimated for surface area calculations using a digital caliper with 0.01 mm precision.

A Discovery TGA-MS (TA Instruments, USA) with open faced HT Pt pans was used to perform constant heating TGA experiments connected to a mass spectrometer (MS) in order to further characterize the mass loss events. A nitrogen gas purging at a flow rate of 25 mL min^−1^ was used for all samples in a non-pressurized furnace. The pans were loaded with an arbitrary amount of compound between 1 and 3 mg. The instrument requires small sample sizes due to the sensitive thermal ionization filament, although many runs have indicated a high level of precision. Pans were torched in an open flame after each sample and tared. Samples were dried in ambient open air for 1 h prior to running.

The TRIOS software was used to analyze derivatives of TGA curves. The sample was heated at 20 °C min^−1^ up to 600 °C in the proprietary Hi-Res^™^ TGA programming. The program heats at constant rate until a weight loss event greater than the desired sensitivity begins. The differential TG curve is taken with respect to temperature due to the varying heating rates of the program. The Discovery TGA-MS is coupled to a thermal ionization filament with mass spectrometry capabilities up to 300 amu.

The melting point of each of the thirteen samples is taken to verify the phase change of the sample at increasing temperatures. Four capillary tube samples of each compound were run simultaneously using a MP50 Melting Point System (Mettler Toledo, USA). The samples are run at 10 °C min^−1^ and with video recording of the samples. Qualitative assessment of the melting point is completed with 0.5 °C margin of uncertainty at the temperature where each sample begins and completes melting.

Elemental analysis was performed by Altantic Microlab in Norcross, GA. Infrared spectra were recorded using a Perkin Elmer FT-attenutaed total reflectance—infrared spectrometer (FT-ATR-IR) spectrum 100 instrument in the range from 4000 to 550 cm^−1^. Mass spectra were recorded using a GBC 9000 Opti-mass inductively coupled plasma time-of-flight mass spectrometer (ICP–TOF–MS) for detection of the RE ions. Mass fragments were analyzed using a HP 5973 inert Mass Selective Detector (EI-MS). NMR data for H and F were obtained using a Varian NMR system at 500 MHz, using 1,4-dioxane_d-99 %_ (Cambridge Isotopes) as a solvent. Single crystal X-ray diffraction data was collected on a Bruker SMART APEXII three circle diffractometer equipped with a CCD area detector and operated at 1800 W power (45 kV, 40 mA) to generate Mo Kα radiation (*λ* = 0.71073 Å). The incident X-ray beam was focused and monochromated using Bruker Excalibur focusing optics. Single crystals were mounted on nylon CryoLoops (Hampton Research) with Paratone-N (Hampton Research) and frozen at −100 and −173 °C, respectively. Initial scans of each specimen were taken to obtain preliminary unit cell parameters and to assess the mosaicity (i.e. breadth of spots between frames) of the crystal to select the required frame width for data collection. For all cases frame widths of 0.5° were judged to be appropriate and full hemispheres of data were collected using the Bruker APEX2 software suite to carry out overlapping *φ* and *ω* scans at detector setting of 2*θ* = 28°.

## Results and discussion

Table 1 below shows the sublimation and vapor pressure data of the thirteen chelates. The regression data is for the trend line of the seven data points of the logarithmic equation, as described earlier. The mass loss rates were found by line fitting each isotherm on the weight versus time graph. Each isotherm exhibited strong linearity (*R* ^2^ > 0.99). Also tabulated is the average sample weight percent lost over the course of the analyzed isotherms for each sample, most of which started the first isotherm with >90 wt%. It has been found that weight loss of about 25 % corresponds to a change in surface area of <15 % [23]. The vapor pressure at a standard temperature of 150 °C is calculated for each chelate using the same Langmuir equation, calibrated to the standards.

**Table 1:** Thermodynamic results on Ln[hfac]_4_ compounds

Compound	Color	*mp* (°C)	Range (°C)	∆*H*_sub_ (kJ mol^−1^)	±*σ* (kJ mol^−1^)	∆ wt%	Pv (150 °C) (atm)
La[hfac]_4_	White	82–89	120–150	137.52	3.79	39.98	0.00028
Pr[hfac]_4_	Green	172–180	115–145	159.97	5.65	44.67	0.00062
Nd[hfac]_4_	Rreddish blue	188–197	126–150	193.81	5.84	35.94	0.00021
Sm[hfac]_4_	Yellow white	180–193	110–140	99.36	3.05	20.20	0.00026
Eu[hfac]_4_	Yellow white	200–208	115–145	86.04	3.29	24.76	0.00015
Gd[hfac]_4_	White	217–220	125–155	103.88	3.60	35.72	0.00011
Tb[hfac]_4_	White	214–218	125–155	103.91	3.64	39.15	0.00013
Dy[hfac]_4_	Yellow white	196–205	115–130	150.19	10.99	15.00	0.00051
			140–155	56.54	7.75	52.87	0.00010
Ho[hfac]_4_	Red	170–180	112–136	105.81	6.07	30.66	0.00025
Er[hfac]_4_	Reddish white	218–226	120–150	121.66	4.61	23.75	0.00015
Tm[hfac]_4_	Yellow white	214–217	125–155	95.12	4.27	29.41	0.00012
Yb[hfac]_4_	Yellow white	212–215	120–150	114.41	4.66	42.07	0.00023
Lu[hfac]_4_	White	194–198	115–145	123.42	4.62	33.29	0.00029

The measured range of isotherms was chosen based on initial trial runs, as well as analysis of the TGA-MS and melting point data. The initial data noted the rate of volatilization, as temperatures too low would result in little to no vaporization of the sample (low mass loss rates) and temperatures too high resulted in excess volatilization and non-negligible change in surface area. The ideal isotherm temperatures were high enough as to cause volatilization, yet not high enough as to cause degradation and large variations in surface area, covering the onset of the major weight loss event. Previous studies have used starting sample weights above 15 mg, and this would obviously provide less of a drastic change in surface area. As the sample weight decreases, the surface area decreases proportionally, and thus the calculated mass loss rate would be lower than that if the surface area was maintained constant throughout. In certain cases, the data point for the last isotherm was neglected if it provided a considerable decline in R-squared value for Eq. (4).

As mentioned earlier, three samples necessitated a change in the isothermal scheme. For the neodymium and holmium analog, the major sublimation event appeared to be over a shorter temperature range than 30 °C, and to ensure enough data points covered this event range, the measured range was shortened to 24 °C, by using isotherms every 4 °C instead of every 5 °C. Still using 7 isotherms for 5 min each, the data improved in linearity. It can be seen that the dysprosium chelate has two sets of sublimation data, corresponding to the two major weight loss events during sublimation, described later in this chapter. The dysprosium analog sublimes in a two-step process, with a 15 wt% loss event followed by a brief period of no weight loss, and then immediately followed by a 50 wt% loss event. The sample was reevaluated by running the isotherm scheme for each step over 15 °C ranges, as noted in Table 1. The isotherms were taken every 3 °C, and still for 5 min each, but with only 6 isotherms per step.

**Fig. 3:**
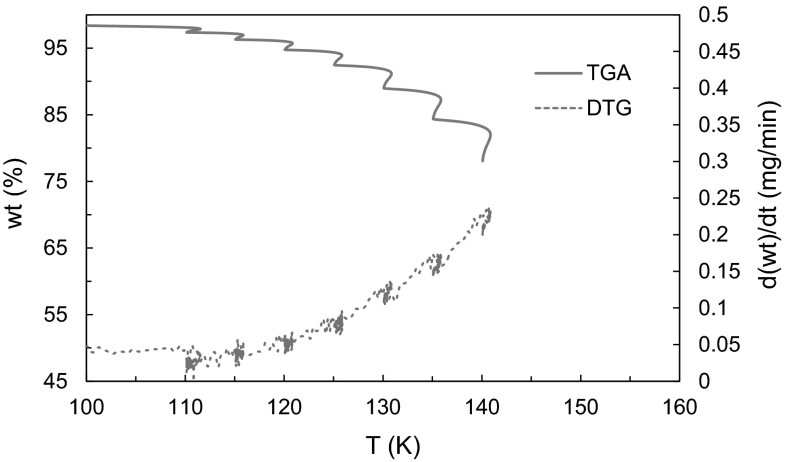
Isothermal jump TGA–DTG spectrum for Sm[hfac]_4_

The first isotherm often results in larger than expected mass loss rate, and thus does not fit linearly with the other data points. This is likely due to the transition in the sample’s vaporization from constant heating to isothermal heating. Additionally, because some samples began isothermal heating at 110 °C, mass loss during the first isotherm may be indicative of water loss as well as sample sublimation. Strong linearity is typically seen starting with the second isotherm up through the sixth isotherm. The set of isotherms with the highest *R* ^2^ value and with the most data points was chosen; often this was isotherms two through six. A typical TGA-DTG diagram with the isothermal jumps is seen in Fig. 3.

The vaporization coefficient α was calculated using standards of Zr[hfac]_4,_ Zr[tfac]_4,_ Zr[acac]_4,_ Cu[hfac]_2,_ Cu[tfac]_2_ and Cr[hfac]_3_ obtained from Strem Chemicals Inc (MA, USA). The same isothermal scheme as mentioned above was used and the literature value of vapor pressure and measured range were taken from literature [15]. The data produced very high precision and linearity and the calculated value of sublimation enthalpy was very close in value to the reported literature value. The value of α was calculated using Langmuir’s equation. This standardized value was used to calculate corrected values of vapor pressure for each compound experimented with non-vacuum TGA.

The uncertainty associated with the sublimation enthalpy was calculated by running a Monte Carlo program to solve for the same enthalpy value a large number of times, each calculated based on randomly chosen uncertainties associated with the mass loss rate value at each temperature tested within standard deviation. The mean and standard deviation of the normal distribution for each sublimation enthalpy are tabulated in Table 1.

The adsorption enthalpy tabulated in Table 2 was calculated from Eq. (5) and corresponds to lanthanide chlorides or oxychlorides on a quartz surface. A kinetic model such as a Monte Carlo simulation of single-atom chemistry can predict retention time from adsorption enthalpy and other column specific parameters. To the authors knowledge, this is the second report of enthalpic values for the Ln[hfac]_4_ compounds [13]. Comparing to previous studies, values presented here exhibit stronger precision and linearity. Knowing the relative differences in enthalpy across the series allows for the proper manipulation of chromatographic parameters to experimentally separate each lanthanide complex on a column.

**Table 2:** Adsorption enthalpy and decomposition onset

Compound	−∆*H*_ads_ (kJ mol^−1^)	±*σ* (kJ mol^−1^)	Decomposition onset (°C)
La[hfac]_4_	104.01	6.64	237
Pr[hfac]_4_	117.48	7.38	220
Nd[hfac]_4_	137.79	7.92	224
Sm[hfac]_4_	81.12	6.05	200
Eu[hfac]_4_	73.12	5.96	220
Gd[hfac]_4_	83.83	6.20	210
Tb[hfac]_4_	83.85	6.21	205
Dy[hfac]_4_	111.61	9.20	200
	55.42	7.12	
Ho[hfac]_4_	84.99	6.88	193
Er[hfac]_4_	94.50	6.63	205
Tm[hfac]_4_	78.57	6.27	200
Yb[hfac]_4_	90.15	6.56	200
Lu[hfac]_4_	95.55	6.65	195

Comparing the adsorption enthalpies across the series, it can be seen that there is no overall linear trend, but there is consistent increases in enthalpy between immediate neighbors. The uncertainty values are propagated from the sublimation data through the empirical correlation described earlier. The onset of decomposition is noted in the above table and is useful information when deciding the column temperature of a chromatographic separation.

The 4*f* electrons of the lanthanide series are very close to the nucleus, therefore the increasing nuclear charge across the period corresponds to decreasing ionic radius. This would lead us to assume that the lanthanide chelates decrease sterically across the period and thus are more volatile for the heavier lanthanides. It is important to note that despite this, the spectrochemical series of ligands and metal ions only indicates that the ligand field splitting parameter increases down a group, i.e., volatility decreases, therefore, there is no correlation across a period.

These two points can be made when analyzing the energetics of the lanthanide series, and it is partially why the enthalpic values of the series appear cyclical in nature. It can be hypothesized that the volatilization of the compounds is more due to the chemical properties rather than the physical properties such as size. Physisorption would be more prevalent if the series followed a trend with ionic radius, and although this may exist for parts of the series, chemisorption must play a larger role when differentiating elements directly next to each other. This is important because the effect of physisorption decreases and the effect of chemisorption increases as chromatographic temperatures increase, up to a certain peak temperature. Therefore the differences seen in this data may be exploited in various chromatographic experiments or simulations.

**Fig. 4:**
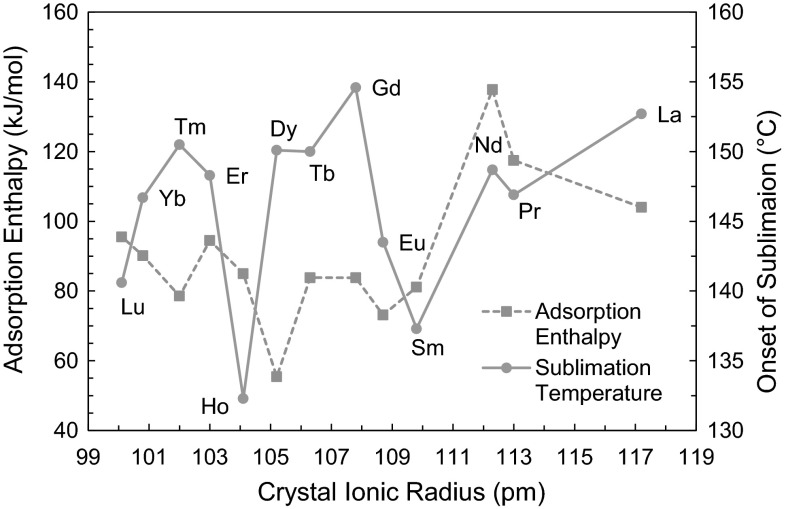
Adsorption enthalpy and sublimation temperature as function of ionic radius

The adsorption enthalpy values and onset of sublimation values are graphed against the crystal ionic radii in Fig. 4 below. The cyclical nature of the series is shown by the decrease in enthalpy magnitude between neighbors of increasing radius. Oppositely, it can be hypothesized that the series overall increases in magnitude of enthalpy between the first group of three and the last group of three. Therefore, both trends are seen in the series, yet in different scales. This would support the lanthanide contraction across the series, yet also indicate immediate neighbors are more influenced by some other factors such as chemical properties or electronic character. Further analysis methods to better understand the coordination of the chelates are necessary to conclude why this trend is seen. The onset of sublimation follows similar trends as adsorption enthalpy, as seen by the figure. Note that the values for Dy refer to the second, larger step of sublimation.

It is also important to note that adsorption and desorption of the compound on a column must occur prior to the onset of decomposition, i.e. an isothermal chromatographic column must run at a temperature below the onset temperature of decomposition. Additionally, a temperature too low may result in large retention times, therefore an ideal temperature for chromatography should be in the sublimation temperature range. Decomposition onset temperatures from the following section are also tabulated in Table 2. It is worth noting that degradation of the compound probably occurs prior to the onset temperature, therefore this value represents the temperatures at which the degraded compounds finally start to sublime or evaporate. As the temperature continues to rise, the remaining solid compound degrades, such as an oxidation reaction, and the sample pan is typically left with a small amount of residue <5 wt% from the end of degradation up to 600 °C.

### TGA-MS and melting point data

**Fig. 5:**
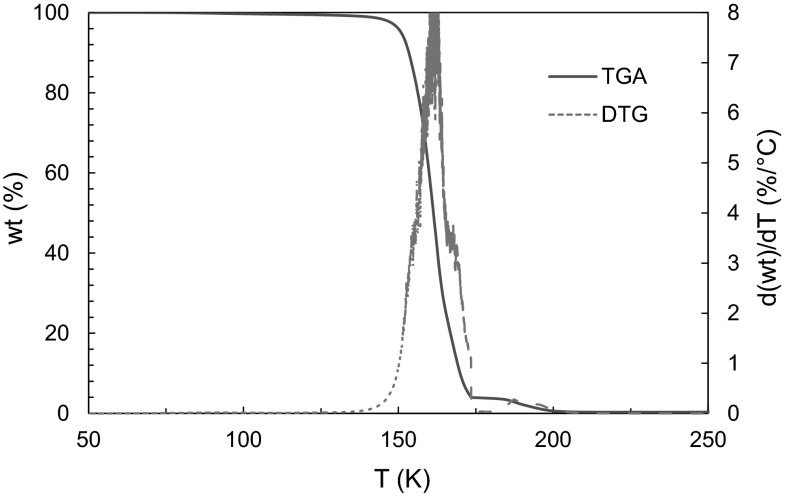
TGA–DTG spectrum for Gd[hfac]_4_

The TGA and DTG spectra for one of the thirteen chelates is shown in Fig. 5. The melting point data is shown in Table 1 above. Each spectra is analyzed for weight loss events which are characterized by percent weight lost during the event, as well as the onset and endset temperature of the event. Most compounds exhibited one major event, and typically accompanied by smaller peaks before and after corresponding to loss of coordinated water molecules or stepwise sublimation. Sublimation is confirmed (as opposed to melting and subsequent evaporation) by comparing melting point analysis, which is run at 10 °C min^−1^. Additional experiments were completed to conclude that this is a thermodynamically similar rate to the HiRes-TGA program at 20 °C min^−1^.

Mass spectrometry data is provided for each mass up to 300 amu. Masses of 18, 32 and 44 are especially important, corresponding to water, oxygen and carbon dioxide, respectively. It is especially important to note that no significant, larger mass peaks are detected during the major weight loss event in any of the spectra. This is noteworthy because it indicates that no mass below 300 amu is detected, and because we know some compound must be vaporizing in the furnace, it can be concluded that the mass of the compound vaporizing is most likely above the mass 300 amu. This would support the understanding that the compound is vaporizing as a chelate, and not as individual components, which is very crucial in understanding the thermal stability of the compounds.

Confirming sublimation of the chelate within these various ranges of temperatures and confirming that the compound is maintain its chelating bond during sublimation are two important conclusions of these experiments. One result of importance is the low melting point of lanthanum hfac, which indicates that sublimation is not occurring during the heating range. This is the only compound of this nature, and therefore the enthalpy calculated in the previous section corresponds to the enthalpy of vaporization, and not the enthalpy of sublimation. This should not be compared to the other results of the series, as the enthalpy of vaporization can represent different thermodynamics, including melting and boiling. Additionally, some chelates degrade as they melt, and therefore the enthalpy of vaporization may not be representative of the energy required for a vapor phase change, which is the goal of these experiments.

Another result of importance is the fact that dysprosium hfac sublimes stepwise in two events successively, losing about 20 % weight first, then about 75 % of the initial weight is lost. This is probably due to dysprosium forming two different chelate forms which sublime at different temperatures. Each event is characterized by its own enthalpy value, yet when comparing to the other chelates in the series, only the second larger event is considered. Holmium also exhibits stepwise sublimation, but with equal weight losses of about 45 % each. Only the first event is characterized with an enthalpic value because of the large mass loss associated with it, unlike that for dysprosium.

### SC-XRD

Determination of crystal structure was only achieved for Gd[hfac]_4_. Following data collection, reflections were sampled from all regions of the Ewald sphere to re-determine unit cell parameters for data integration. Following exhaustive review of collected frames the resolution of the dataset was judged, and, if necessary, regions of the frames where no coherent scattering was observed were removed from consideration for data integration using the Bruker SAINTplus program. Data was integrated using a narrow frame algorithm and was subsequently corrected for absorption. Absorption corrections were performed for both samples using the SADABS program. Space group determination and tests for merohedral twinning were carried out using XPREP. The highest possible space group was chosen.

**Fig. 6:**
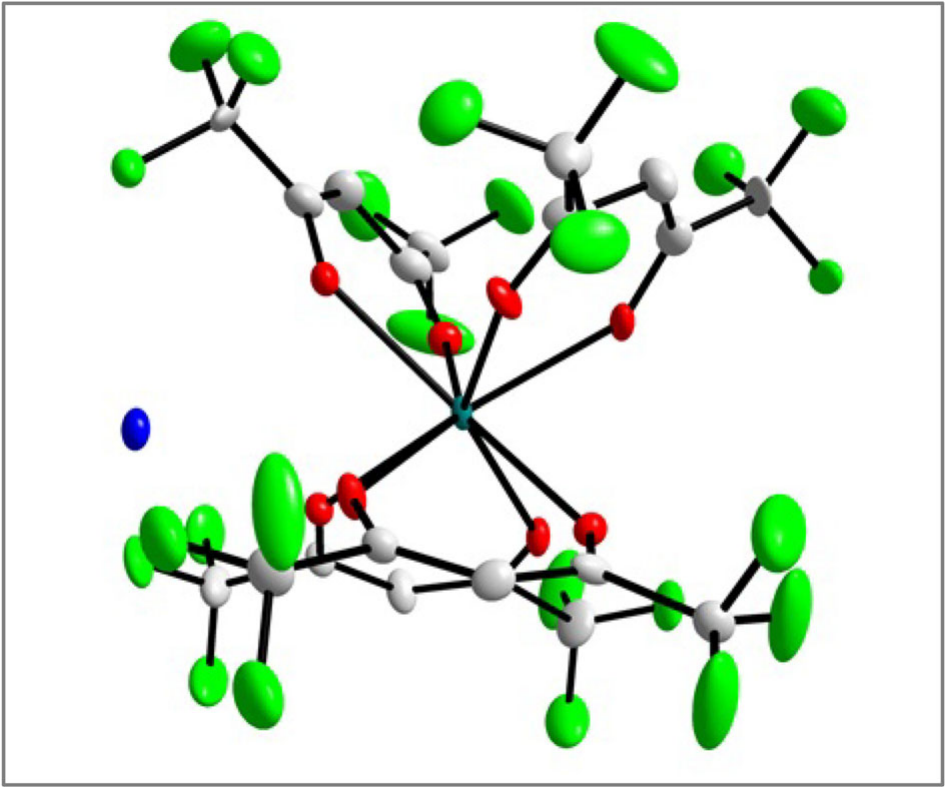
Crystal structure of NH_3_^+^(Gd[hfac]_4_^−^)

The final model was refined anisotropically (with the exception of H atoms). Hydrogen atoms were not placed on solvent molecules due to disorder. The structure was examined using the Addsym subroutine of PLATON4 to assure that no additional symmetry could be applied to the models. The structure arrived at is shown in Fig. 6.

### Elemental analysis

For **1**. Formula: Sm[hfac]_4_. Yield: 35.68 %. Elemental analysis calc’d (%): C 24.37, H 1.75 N 4.06, F 45.80. Found: C 24.3, H 1.87, N 4.06, F N/A. For **2**. Formula: Gd[hfac]_4_. Yield: 65.34 %. Elemental analysis calc’d: C 24.84, H 1.04 N 2.90, F 47.15. Found: C 24.35, H 0.96, N 2.20, F 42.74. For **3**. Formula: Dy[hfac]_4_. Yield: 73.77 %. Elemental analysis calc’d: C 23.71, H 1.93, N 3.95, F 45.00. Found: C 24.45, H 2.08, N 3.87, F 46.10. For **4**. Formula: Tm[hfac]_4_. Yield: 58.17 %. Elemental analysis calc’d: C 23.42, H 1.18, N 2.43, F 44.46. Found: C 24.18, H 1.18, N 2.50, F N/A.

### FTIR

For **1.** (ATR cm^−1^): 3184 (br), 1644 (w), 1563 (w), 1538 (w), 1440 (m), 1252 (m), 1194 (m), 1179 (m), 1130 (br, s), 805 (w), 744 (m). For **2**. (ATR cm^−1^): 3127 (br), 3040 (br), 1645 (s), 1611 (w), 1563 (w), 1537 (m), 1502 (w), 1472 (w), 1405 (m), 1349 (w), 1253 (s), 1201 (s), 1136 (s), 1096 (s), 804 (s), 768 (w), 744 (s), 752 (w), 661 (s). For **3**. (ATR cm^−1^) 3211 (br), 1645 (w), 1564 (w), 1535 (w), 1459 (m), 1253 (m), 1196 (s), 1177 (s), 1123 (s), 800 (m), 738 (m). For **4**. (ATR cm^−1^) 3149 (br), 1649 (w), 1564 (w), 1537 (w), 1473 (m), 1251 (m), 1203 (s), 1177 (s), 1132 (s), 804 (m), 744 (m).

### NMR

For **1**: 1H NMR (500 MHz, 1,4-Dioxane-d8) δ 7.26 (s, 1H), 5.34 (s, 1H), 2.64 (s, 2H). 19F NMR (470 MHz, dioxane) δ−76.50, −76.98, −77.41. For **2**: 1H NMR (500 MHz, dioxane) δ 6.91, 2.37. 19F NMR (470 MHz, dioxane) δ−78.02, −79.70, −80.93. For **3**: 1H NMR (500 MHz, dioxane) δ 1.03, 0.97. 19F NMR (470 MHz, dioxane) δ−71.83, −75.13, −76.22, −77.42, −78.24. For **4**: 1H NMR (500 MHz, 1,4-Dioxane-d8) δ 4.72 (s, 1H). 19F NMR (470 MHz, dioxane) δ−77.49, −108.66, −109.94.

## Conclusions

The sublimation enthalpies and sublimation temperatures for a series of metal β-diketonate complexes, specifically Ln[hfac]_4_, were determined using thermogravimetric analysis. No linear trend is seen across the series of lanthanoids, although the values display a cyclical nature with increases in value between immediate neighbors. Small amounts of coordinated molecules and byproducts are found by analysis of the regions before and after the isotherms by TGA-MS. Melting point analysis was used to confirm sublimation. Mass spectroscopy data was used to confirm the stability of the chelate as it volatilizes. Further characterization of four of the chelates is achieved via SC-XRD, Elemental Analysis, FTIR and NMR, which is provided in the supplementary information.

A significant improvement to the efficiency of lanthanide separations in a solvent-free environment has been achieved, as was proven by initial results reported by Stratz et al. using these complexes [36]. An empirical correlation to adsorption enthalpy was used to estimate the thermodynamics of chromatographic separations of lanthanide oxides on a quartz column. Initial gas-phase separation results point toward realistic and decisively proficient separations using a gas chromatography mass spectrometer system using the 1,1,1,5,5,5-hexafluoroacetylacetone ligand. Resulting thermodynamic parameters yield the ability to optimize temperature and pressure settings for enhanced time-resolved separations when attempting to separate mixtures of several lanthanide complexes. This ability is not only useful in applied nuclear forensics applications, but it translates to potential efficiency and sustainability improvements within the commercial RE industry.

Future work may involve other methods of thermal analysis such as differential scanning calorimetry to further characterize the volatility and stability of these compounds. In addition to thermal methods, UV–Vis spectroscopy can be used to characterize the complex formation of the chelates, specifically the compounds with multiple coordination types. Finally, the residue after thermal analysis can be characterized to confirm the resulting solid, believed to be oxide.

## Electronic supplementary material

Below is the link to the electronic supplementary material.

Supplementary material 1 (DOCX 383 kb)
