# Supplementary material for: Characterization and thermogravimetric analysis of lanthanide hexafluoroacetylacetone chelates
Source: J Radioanal Nucl Chem. 2016 Aug 30;311(1):617–26. doi: 10.1007/s10967-016-5005-0 (PMC5219043; doi:10.1007/s10967-016-5005-0)
Supplement: Supplementary file 1 — Supplementary material 1 (DOCX 383 kb) [file 10967_2016_5005_MOESM1_ESM.docx]

Supplementary information

**ICP-TOF-MS**

Prepared samples of each of the four Ln[hfac]_3_ compounds were analyzed with an inductively-coupled plasma time-of-flight mass spectrometer for RE metal content.


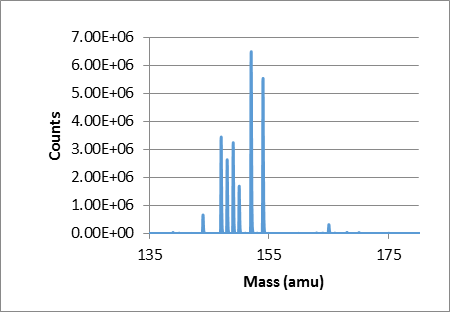


**Fig. 1** Sm[hfac]_4_ ICP-TOF-MS


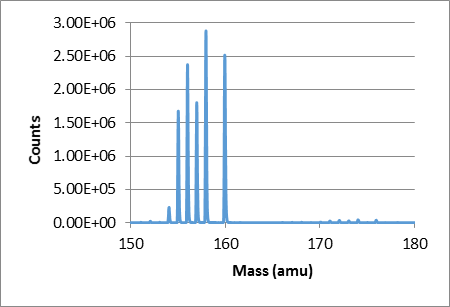


**Fig. 2** Gd[hfac]_4_ ICP-TOF-MS


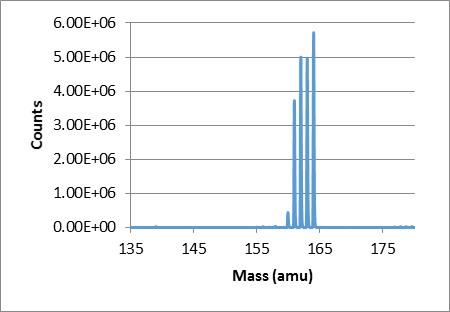


**Fig. 3** Dy[hfac]_4_ ICP-TOF-MS


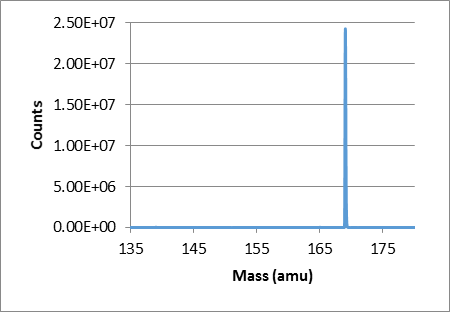


**Fig. 4** Tm[hfac]_4_ ICP-TOF-MS

**FTIR**

**
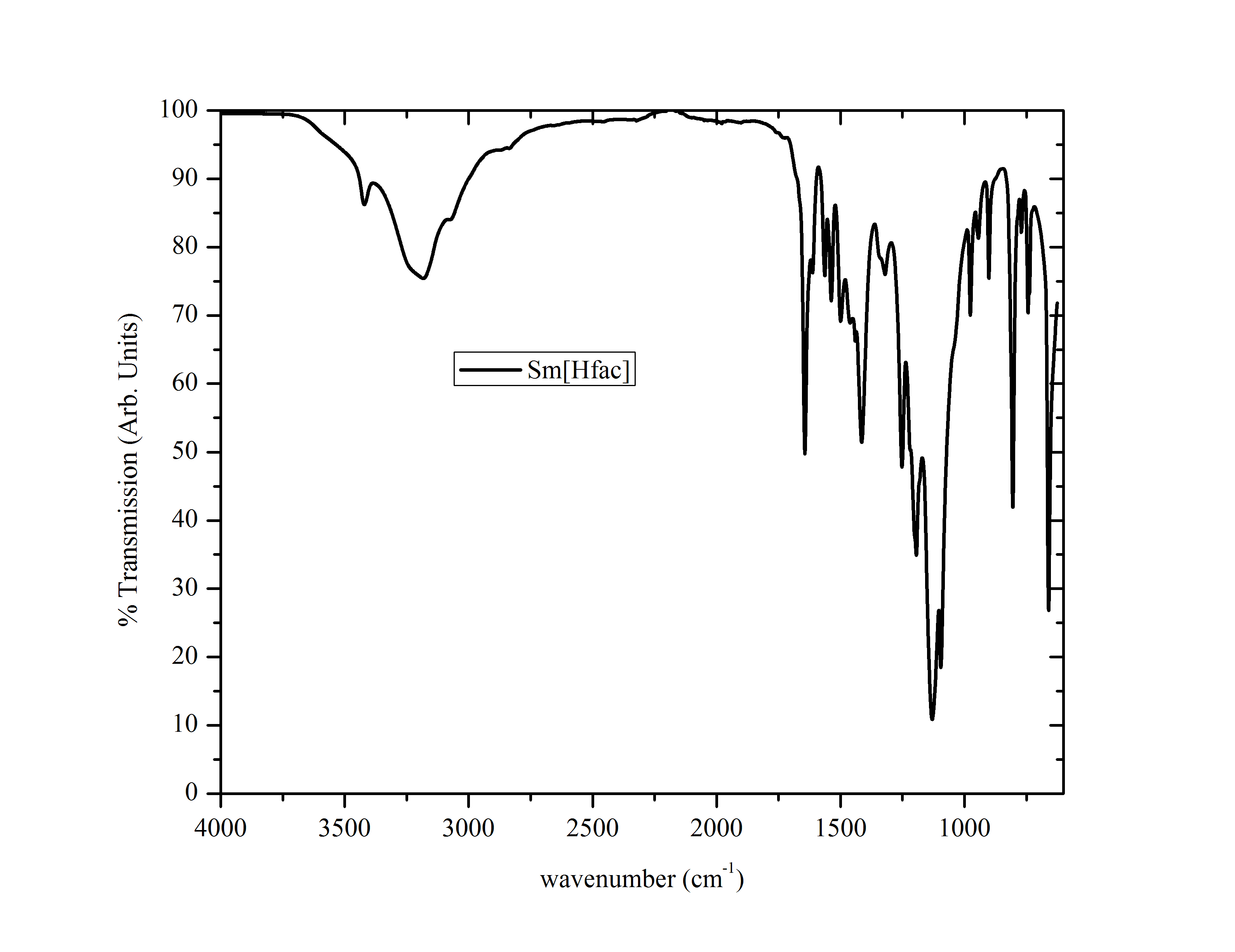
**

**Fig. 11** Complex **1** FTIR Data

**
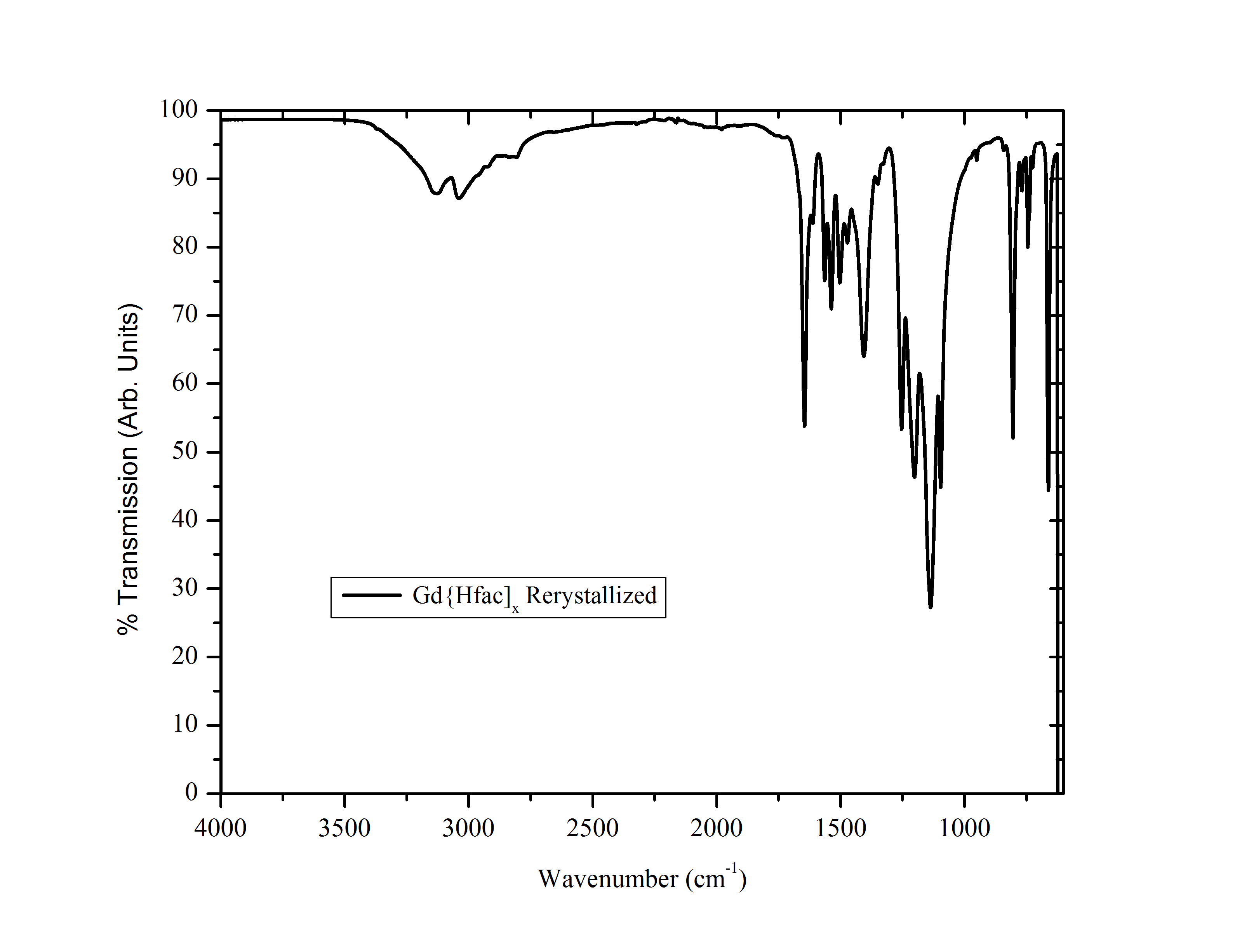
**

**Fig. 12** Complex **2** FTIR Data

**
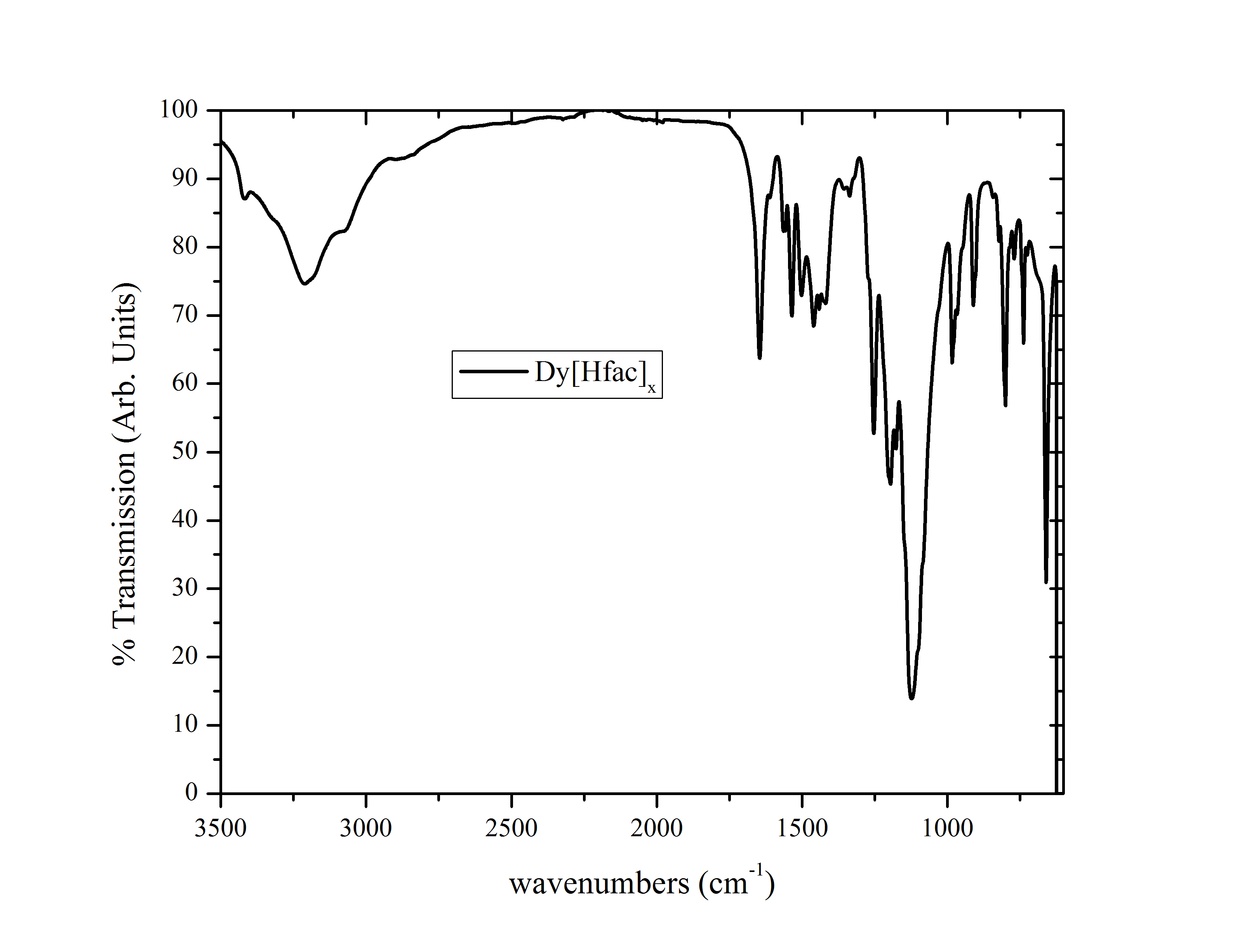
**

**Fig. 13** Complex **3** FTIR Data

**
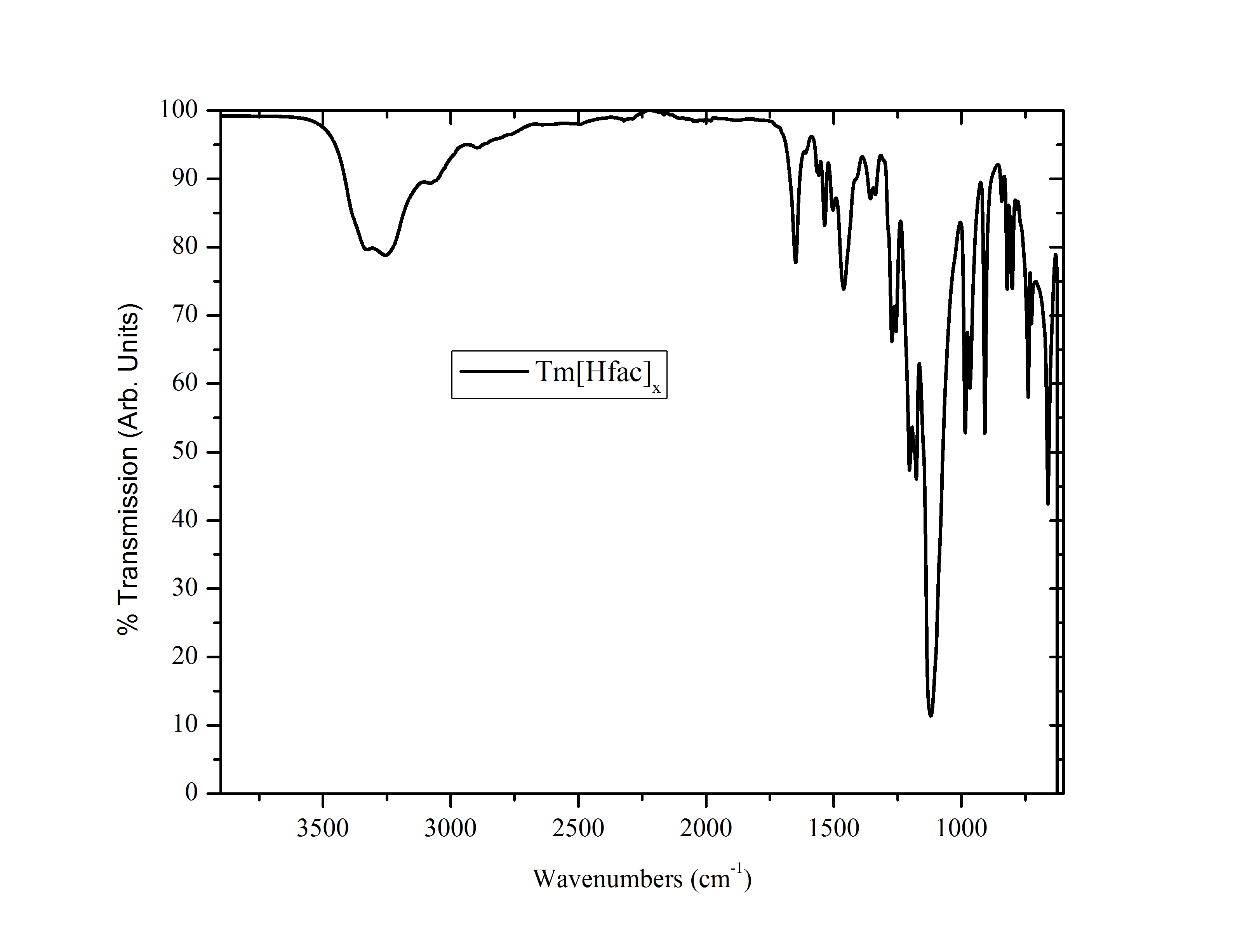
**

**Fig. 14** Complex **4** FTIR Data

**NMR**


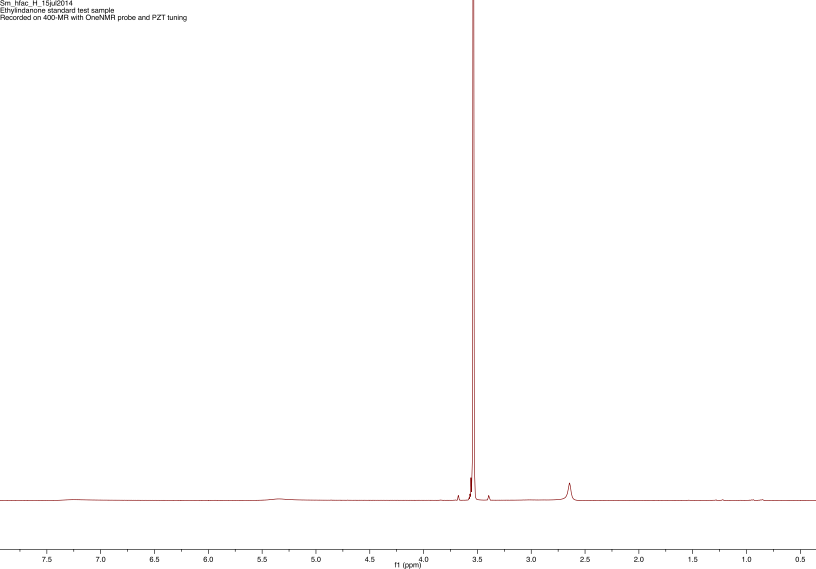


**Fig. 15** H-1 NMR of Complex 1

**
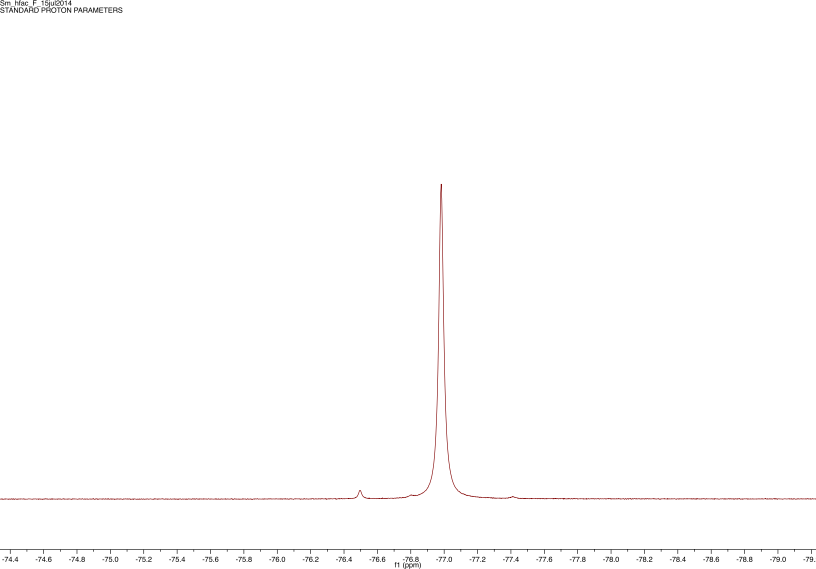
**

**Fig. 16** F-19 NMR of Complex 1


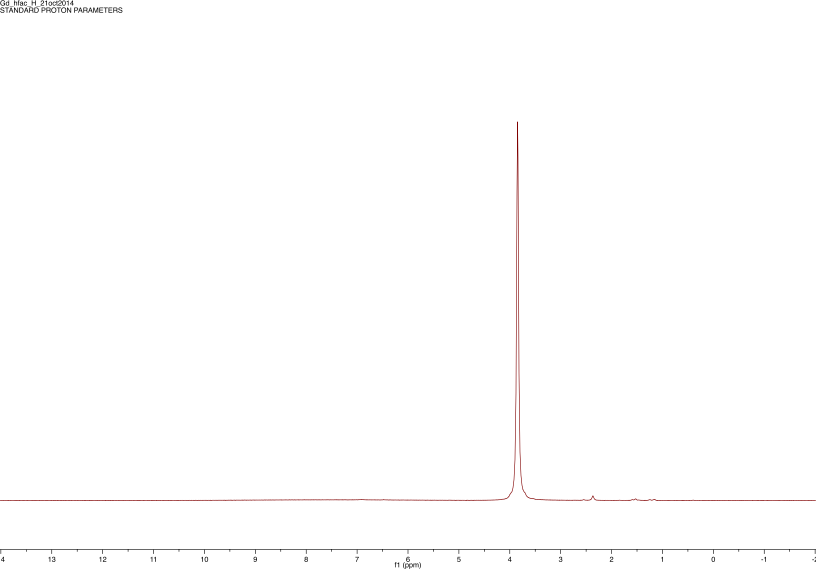


**Fig. 17** H-1 NMR of Complex 2


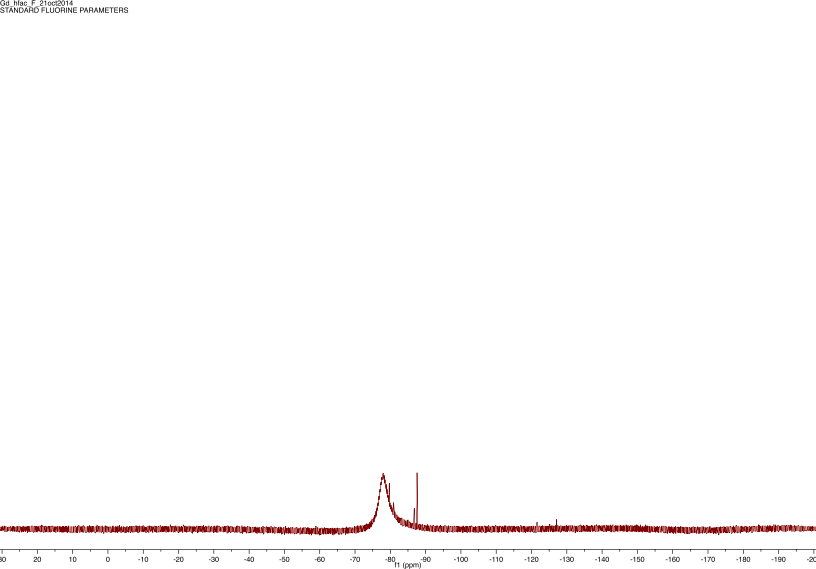


**Fig. 18** F-19 NMR of Complex 2


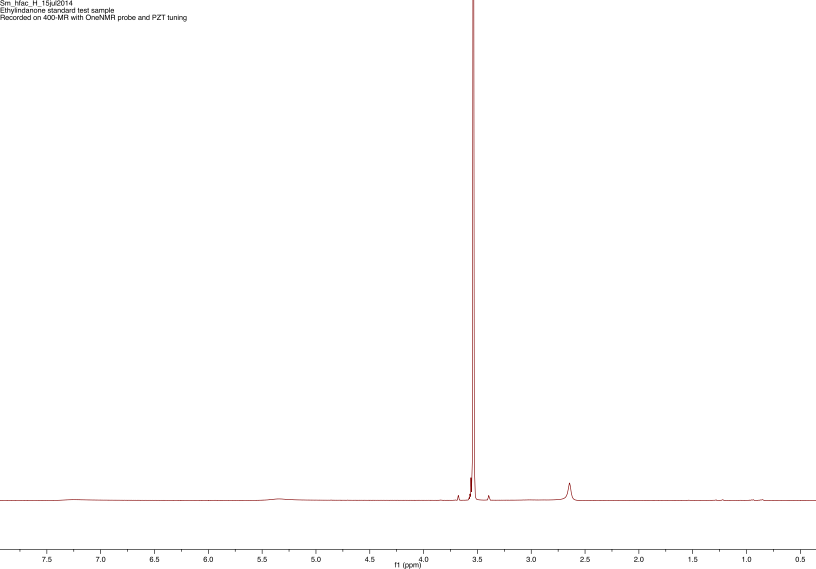


**Fig. 19** H-1 NMR of Complex 3


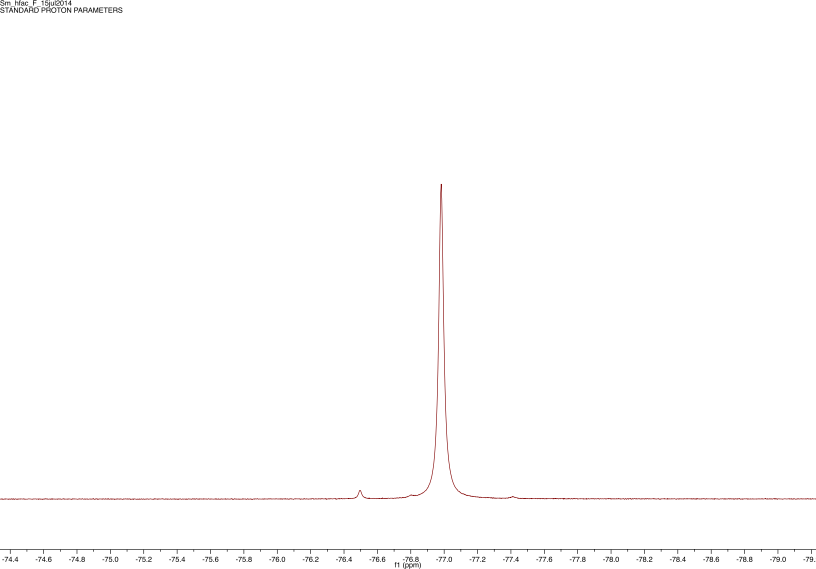


**Fig. 20** F-19 NMR of Complex 3


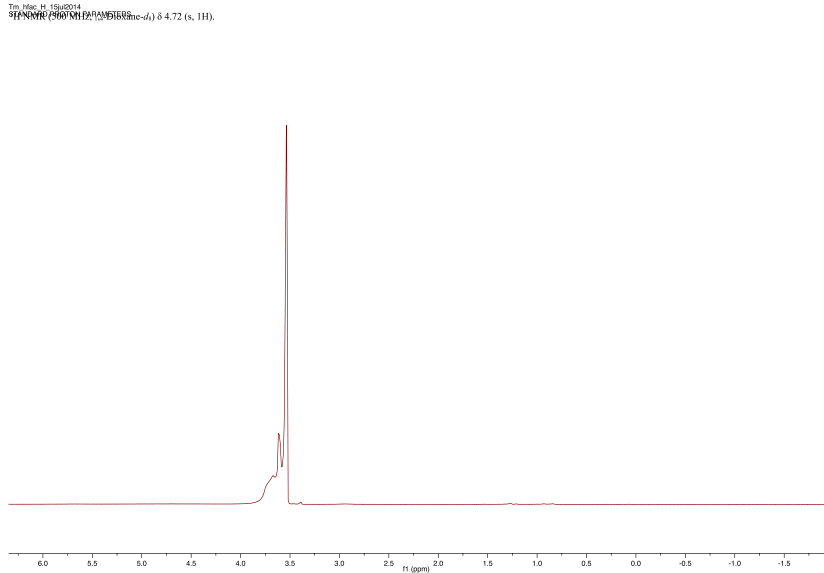


**Fig. 21** H-1 NMR of Complex 4


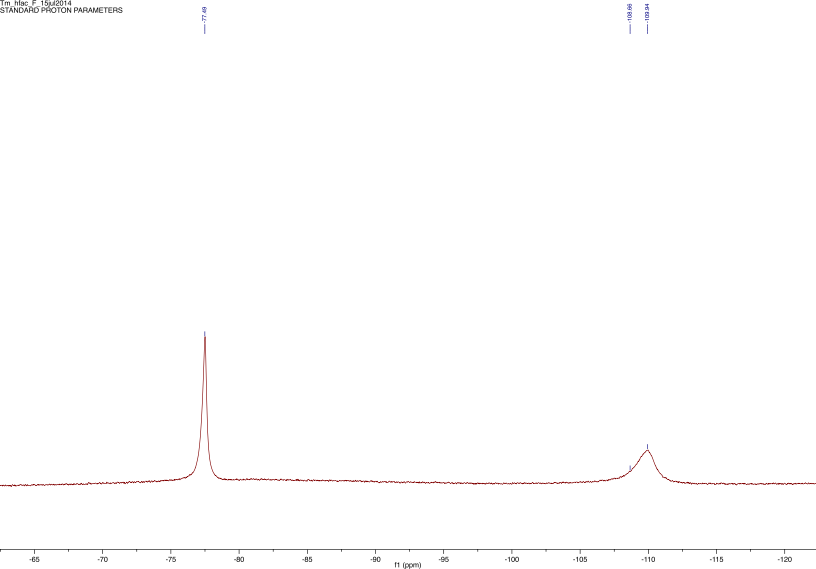


**Fig. 22** F-19 NMR of Complex 4
